# Molecular mechanisms of long non-coding RNAs in anaplastic thyroid cancer: a systematic review

**DOI:** 10.1186/s12935-020-01439-w

**Published:** 2020-07-29

**Authors:** Hilda Samimi, Sayed Mahmoud Sajjadi-Jazi, Soroush Seifirad, Rasha Atlasi, Habibollah Mahmoodzadeh, Mohammad Ali Faghihi, Vahid Haghpanah

**Affiliations:** 1 Endocrinology and Metabolism Research Center, Endocrinology and Metabolism Clinical Sciences Institute, Tehran University of Medical Sciences, Tehran, Iran; 2 Cell Therapy and Regenerative Medicine Research Center, Endocrinology and Metabolism Molecular-Cellular Sciences Institute, Tehran University of Medical Sciences, Tehran, Iran; 3 Division of Cardiology, Department of Medicine, Beth Israel Deaconess Medical Center, Harvard Medical School, PERFUSE Study Group, Boston, MA USA; 4 Evidence Based Practice Research Center, Endocrinology and Metabolism Clinical Sciences Institute, Tehran University of Medical Sciences, Tehran, Iran; 5 Department of Surgery, Iranian National Cancer Institute, Imam Khomeini Complex, Tehran University of Medical Sciences, Tehran, Iran; 6 Persian BayanGene Research and Training Center, Dr. Faghihi’s Medical Genetic Center, Shiraz, Iran; 7 Department of Psychiatry and Behavioral Sciences, University of Miami Miller School of Medicine, Miami, USA; 8 Personalized Medicine Research Center, Endocrinology and Metabolism Clinical Sciences Institute, Tehran University of Medical Sciences, Tehran, Iran; 9 Endocrinology and Metabolism Research Center (EMRC), Dr. Shariati Hospital, North Kargar Ave., Tehran, 14114 Iran

**Keywords:** Long non-coding RNA, Anaplastic thyroid cancer, Cancer stem cell, Systematic review, Tumor suppressor, Oncogene

## Abstract

**Background:**

anaplastic thyroid cancer (ATC) is one of the most lethal and aggressive cancers. Evidence has shown that the tumorigenesis of ATC is a multistep process involving the accumulation of genetic and epigenetic changes. Several studies have suggested that long non-coding RNAs (lncRNAs) may play an important role in the development and progression of ATC. In this article, we have collected the published reports about the role of lncRNAs in ATC.

**Methods:**

“Scopus”, “Web of Science”, “PubMed”, “Embase”, etc. were systematically searched for articles published since 1990 to 2020 in English language, using the predefined keywords.

**Results:**

961 papers were reviewed and finally 33 papers which fulfilled the inclusion and exclusion criteria were selected. Based on this systematic review, among a lot of evidences on examining the function of lncRNAs in thyroid cancer, there are only a small number of studies about the role of lncRNAs and their molecular mechanisms in the pathogenesis of ATC.

**Conclusions:**

lncRNAs play a crucial role in regulation of different processes involved in the development and progression of ATC. Currently, just a few lncRNAs have been identified in ATC that may serve as prognosis markers such as GAS5, MIR22HG, and CASC2. Also, because of the dysregulation of Klhl14-AS, HOTAIRM1, and PCA3 during ATC development and progression, they may act as therapeutic targets. However, for most lncRNAs, only a single experiment has evaluated the expression profile in ATC tissues/cells. Therefore, further functional studies and expression profiling is needed to resolve this limitation and identify novel and valid biomarkers.

## Background

Thyroid cancer is the most frequent malignant neoplasm of the endocrine system [1]. Although the greater part of thyroid cancers have a good prognosis, some of these are correlated to further aggressive clinical manner [2]. Anaplastic thyroid cancer (ATC) is the most lethal and aggressive thyroid cancer accounting 1–3% and its generally survival rate is just 3–6 months following initial diagnosis [3].

In thyroid cancer, the majority of genetic alterations are directly associated with dysregulation of MAPK and PI3K/AKT signaling pathways for instance point mutations in *BRAF* and *RAS* genes, that have an important role in the modulation of cell growth, survival, and proliferation [4–6]. Also, epigenetic changes have a key role in the alterations of gene expression pattern in thyroid cancer [7].

In addition to genetic and epigenetic events, other biological mechanisms including non-coding RNAs (ncRNAs) play an important role in regulation of approximately all steps of cancer progression such as cell growth, epithelial to mesenchymal transition (EMT), and multidrug resistance (MDR) [6, 8]. Based on their size, ncRNAs are usually classified as small ncRNAs incuding miRNAs, siRNAs, piRNAs, etc. and long non-coding RNAs (lncRNAs) with the length of longer than 200 nucleotides [8]. In contrast to small ncRNAs, lncRNAs are much less recognized relating to their roles in tumorigenesis and cancer progression, especially in ATC [9, 10].

In this article, we conducted a systematic review to evaluate the role of lncRNAs in ATC and discuss their molecular mechanisms in the pathogenesis of ATC and importance as potential diagnostic and prognostic biomarkers and therapeutic molecular targets for ATC.

## Method

The main databases searched were “Scopus”, “Web of Science (ISI)”, “PubMed”, and “Embase”. Furthermore, to find more evidences, other sources such as “Google Scholar” were reviewed. The main search keywords were “long non-coding RNA”, “noncoding RNA”, “thyroid cancer” and “anaplastic thyroid cancer”, and their logical combinations, related words and phrases, Medical Subject Headings (MeSH) terms, EBSCO thesaurus terms and Emtree terms. The search was restricted to the articles published in English language.

The inclusion criteria were studies which assessed the role of lncRNAs in thyroid cancer from 1990 to February 2020. The exclusion criteria were defined as articles which focused only on the role of lncRNAs in thyroid cancer other than ATC and non-English language studies. The titles and abstracts of articles were reviewed at once for the inclusion and exclusion criteria. In the next step, the full texts of the selected articles were re-evaluated to ensure they meet the eligibility criteria. Finally, to increase the comprehensiveness of the study, the reference lists of the selected articles were reviewed to identify other relevant studies.

## Results

**Fig. 1:**
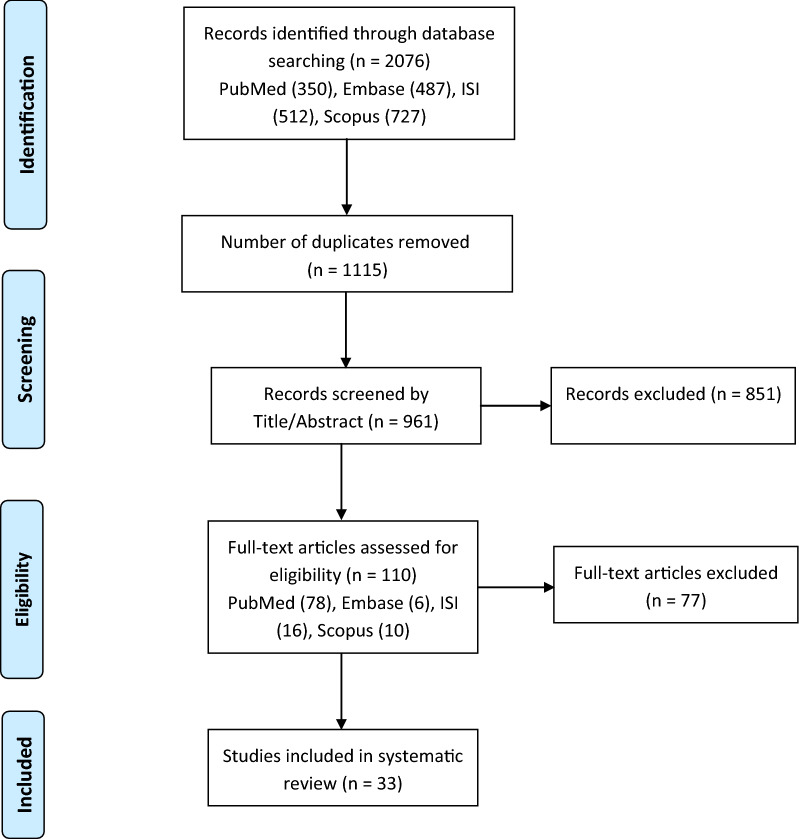
Flowchart of the systematic review (PRISMA)

In the systematic search, 2076 published articles were identified. After removing the duplicates, 961 papers remained, which were reviewed by title/abstract and 851 articles were excluded because of irrelevant contents, lack of details, review articles and conference abstract as well as non-English papers. Finally, among the remaining 110 studies, 33 papers were found to fulfill the eligibility criteria by a full-text review and were selected for a systematic review. Figure 1 demonstrates the search flowchart of evidence acquisition according to the preferred reporting items for systematic reviews and meta-analyses (PRISMA) method. Based on our literature review, among a lot of evidences on examining the function of lncRNAs in thyroid cancer, there are only a small number of studies about the role of lncRNAs and their molecular mechanisms in the pathogenesis of ATC. All of the lncRNAs demonstrated to be involved in the development and progression of ATC are summarized in Table 1.

**Table 1:** LncRNAs associated with ATC

Author/year	LncRNA	Chromosomal location	Sample		Expression	Summary of findings	Refs.
			Cell line/tumor tissue				
Kim/2016	LOC100507661	3q26.2	C643 8505C	–	Up	↑ Cell proliferation, migration, and invasion^a^	[11]
Gu/2017	GAS5	1q25.1	–	33	Down	↑ Cell cycle progression and proliferation probably by upregulating of CDK6 ↓ Survival rate^b^ Poor prognosis	[12]
Zhang/2019	H19	11p15.5	8505C SW1736 KAT18 CAL62	19	Up	↑ Proliferation, migration, and invasion ↓ Apoptosis	[13]
Wang/2018	UCA1	19p13.12	SW1736 KAT18	8	Up	↑ cell viability, proliferation, migration, and invasion via binding to miR-135a and regulation of c-myc expression	[14]
Wang/2019	HOTAIRM1	7p15.2	–	10	Up	↑ HOTAIRM1 expression during thyroid cancer development and progression	[15]
Wang/2019	PCA3	9q21.2	–	10	Up	↑ PCA3 expression during thyroid cancer development and progression	[15]
Chen/2019	HCP5	6p21.33	SW1736 8305C ARO FRO	19	Up	↑ Cell viability and ↓ Apoptosis by regulating the expression of miR-128-3p	[16]
Credendino/2019	KLHL14-AS (Klhl14)^c^	18q12.1	8505C CAL62	9	Down	↑ Cell viability and ↓ Cpoptosis and differentiation by binding to miR-182-5p and miR-20a-5p and regulation of BCL2 and PAX8 expression	[17]
Du/2018	Linc00210	1q41	8505C	N/A	Up	↑ Proliferation, migration, and invasion via binding to miR‐195‐5p and upregulation of IGF1R expression	[18]
Hou/2018	TNRC6C-AS1	17q25.3	ARO Hth74	N/A	Up	↑ Proliferation, migration, and invasion via binding to miR‐129‐5p and upregulation of UNC5B expression	[19]
Li/2017	AK139328	N/A	8505C	N/A	Up	↑ Cell viability, invasion, and cell cycle progression	[20]
Song/2017	LINC00312	3p25.3	8505C	13	Down	↑ Proliferation, invasion, and migration by regulating of miR-197-3p expression and its target p120	[21]
Min/2018			8505C	N/A	Down	↑ Proliferation and invasion by suppressing PI3K/AKT signaling pathway	[22]
Liu/2018	XIST	Xq13.2	SW1736 KAT18	–	Up	↑ Proliferation through binding to miR-34a and upregulation of MET expression ↑ MET/PI3K/AKT signaling pathway	[23]
Chen/2019	SNHG7	9q34.3	CAL62	N/A	Up	↑ Proliferation and cell cycle progression	[24]
Liu/2018	SNHG15	7p13	8505C	N/A	Down	↑ Proliferation, migration, and invasion via upregulating miR-510-5p	[25]
Han/2019	ZFAS1	20q13.13	CAL62	20	Up	↑ Proliferation and cell cycle progression	[26]
Qin/2019	MIR22HG	17p13.3	–	11	Down	↓ MIR22HG is correlated to higher age, lymph node metastasis, and advanced TNM stages ↑ MIR22HG expression is associated with longer overall and disease-free survival time in thyroid cancer patients.	[27]
Xiong/2017	CASC2	10q26.11	8505C CAL62 HTH-83	N/A	Down	?↓ CASC2 expression is correlated with multifocality and advanced TNM stages ↑ CASC2 expression decreases the proliferation and arrests cell cycle at G_0_/G_1_	[28]
Yan/2019	NEAT1	11q13.1	8505C SW1736	26	Up	↓ Cisplatin-resistance through regulating of miR-9-5p/SPAG9 axis	[29]
LncRNAs related to CSCs properties^d^							
Hardin/2018	ROR	18q21.31	THJ-16T	–	Up	↑ CSC properties probably by modulating of OCT4, SOX2, and Nanog expression ↑ ROR expression in CSC clones of ATC line THJ-16T compared to the parental line ↑ Stem cell marker SOX2 and EMT marker SLUG	[30]
Zhou/2016	PVT1	8q24.21	8505C	7	Up	↑ Cell cycle progression and proliferation by modulating of cyclin D1 and TSHR expression and also EZH2 recruitment	[31]
Hardin/2018			THJ-16T	–	Down	↓ PVT1 expression in CSC clones of ATC line THJ-16T compared to the parental line ↑ Stem cell marker SOX2 and EMT marker SLUG	[30]
Hardin/2018	HOTAIR	12q13.13	THJ-16T	–	Down	↓ HOTAIR expression in CSC clones of ATC line THJ-16T compared to the parental line ↑ Stem cell marker SOX2 and EMT marker SLUG	[30]
Huang/2016			SW1736 KAT18	–	UP	↑ Proliferation and invasion via upregulating of IQGAP1 expression	[32]
Zhang/2017			–	35	Down	↓ MALAT1 expression in ATC tissue samples	[33]
Samimi/2019	MALAT1	11q13.1	SW1736 C643	–	Up	↑ Mcl1 and cyclin D1 expression ↓ miR-363-3p expression	[34]
Gou/2019			SW1736 8505C	–	Up	↑ Proliferation, migration, and invasion ↓ Apoptosis and autophagy via binding to miR‐200a‐3p and upregulation of FOXA1 expression	[35]
Hardin/2018			THJ-16T	–	Up	↑ MALAT1 expression in CSC clones of ATC cell line compared to the parental line ↑ Stem cell marker SOX2 and EMT marker SLUG	[30]
Liu/2017	BANCR	9q21.12	8505C	–	Up	↑ Cell growth, colony formation ability, and cell cycle progression via regulating of BANCR/TSHR/CCND1 signaling pathway	[36]
Liu/2019	FOXD2-AS1	1p33	8305C CAL62 BHT101	10	Up	↑ FOXD2-AS1 expression in cell lines and tissues FOXD2-AS1 acts as a ceRNA for miR-7-5p and upregulates TERT expression, which increases the CSCs properties in thyroid cancer cells	[37]
Wang/2018	PTCSC3	14q13.3	8505C	20	Down	↑ CSC properties by upregulating of STAT3, INO80, CD^133^, and ALDH1 ↑ Drug resistance by upregulating of MDR-1 via STAT3/INO80 pathway	[38]
Fan/2013			8505C	–	Down	↓ Apoptosis ↑ Cell growth and cell cycle progression probably by modulating of miR-574-5p	[39]
Lu/2018	MANCR	10p15.1	BHT101	–	Up	↓ Apoptosis ↑ Proliferation, colony formation, cell cycle progression G_0_ to G_1_, and EMT by modulating E-cadherin, N-cadherin, and β-catenin expression	[40]
Pellecchia/2020	PAR5	15q11.2	8505C ACT1 FB-1 FRO	9	Down	↑ Proliferation, colony formation, and EMT through regulating EZH2 recruitment and E-cadherin expression	[41]
Lei/2017	TUG1	22q12.2	SW1736 KAT18	N/A	Up	↑ Proliferation, colony formation, invasion, migration, and EMT through regulating miR-145/ZEB1 signaling pathway	[42]

AKT, AKT serine/threonine kinase; ALDH1, aldehyde dehydrogenase 1; BANCR, BRAF-activated non-protein coding RNA; BCL2, BCL2 apoptosis regulator; CASC2, cancer susceptibility candidate 2; CCND1, cyclin D1; CDK6, cycling-dependent kinase 6; ceRNA, competing endogenous RNA; c-myc, avian myeocytomatosis virus oncogene cellular homolog; CSC, cancer stem cell; EMT, epithelial to mesenchymal transition; EZH2, enhancer of zeste homolog 2; FOXA1, forkhead box protein A1; FOXD2-AS1, FOXD2 adjacent opposite strand RNA 1; GAS5, growth arrest special 5; HCP5, HLA complex P5; HOTAIR, HOX transcript antisense RNA; HOTAIRM1, hox antisense intergenic RNA myeloid 1; IGF1R, insulin like growth factor 1 receptor; INO80, INO80 complex ATPase subunit; IQGAP1, Ras GTPase-activating-like protein; Klhl14, kelch like family member 14; Linc00210, long intergenic non-protein coding RNA 210; LINC00312, long intergenic non-protein coding RNA 312; LncRNA, long noncoding RNA, MALAT1: metastasis-associated lung adenocarcinoma transcript 1; MANCR, mitotically associated long non coding RNA; Mcl1, myeloid cell leukemia 1; MDR-1, multidrug resistance protein 1; MET, MET protooncogene; miR, microRNA; MIR22HG, MIR22 host gene; NEAT1, nuclear paraspeckle assembly transcript 1; OCT4, octamer-binding transcription factor 4; PAR5, Prader Wili/Angelman region RNA 5; PAX8, paired box 1; PCA3, prostate cancer antigen 3; PI3K, phosphatidylinositol-3-kinas; PTCSC3, papillary thyroid carcinoma susceptibility candidate 3; PVT1, plasmacytoma variant translocation 1; ROR, regulator of reprogramming; SNHG15, small nucleolar RNA host gene 15; SNHG7, small nucleolar RNA host gene 7; SOX2, SRY-box transcription factor 2; SPAG9, sperm associated antigene 9; STAT3, signal transducer and activator of transcription 3; TNM, tumor-node-metastasis; TNRC6C-AS1, TNRC6C antisense RNA 1; TUG1, taurine up-regulated gene 1; UCA1, urothelial carcinoma-associated 1; UNC5B, unc-5 netrin receptor B; XIST, X inactive specific transcript; ZEB1, zinc finger E-box binding homeobox 1; ZFAS1, ZNFX1 antisense RNA 1

^a^↑ Increase

^b^↓ Decrease

^c^ In GeneCards database is named Klhl14

^d^Based on the criteria defined in the text

## Discussion

Rising research indicates that lncRNAs have an important function in biology of thyroid cancers. Dysregulation of some lncRNAs is involved in thyroid tumorigenesis and its progression and can be important targets for molecular targeted therapy of thyroid cancers [43]. Although, the number of evidences on lncRNAs expression in thyroid cancer is growing, there are only a few studies about the role of lncRNAs and their molecular mechanisms on ATC. LncRNAs can act as tumor suppressor or oncogene by influencing important processes such as proliferation, invasion, metastasis, cell cycle, and apoptosis as well as cancer stem cell (CSC) properties during ATC tumorigenesis and progression (Tables 2, 3).

### Tumor suppressive lncRNAs in ATC

CASC2 (cancer susceptibility candidate 2) is reported in several cancer types, acting as a tumor suppressor lncRNA with implications for diagnosis, therapy and prognosis. Upregulation of CASC2 decreased the cell proliferation of non-small cell lung cancer, representing that CASC2 can be a potential prognostic marker and therapeutic target in non-small cell lung cancer therapy [44]. Xiong et al. indicated that the low expression of CASC2 is correlated with multifocality and advanced tumor-node-metastasis (TNM) stage. Their findings showed that the expression of CASC2 can be an independent prognostic factor in thyroid cancer patients. In addition, upregulation of CASC2 decreased the proliferation and arrested cell cycle at G_0_/G_1_ phase in ATC cell lines [28]. Further studies are required to determine the CASC2 function as a potential prognostic marker and therapeutic target in ATC.

Guo et al.. demonstrated that GAS5 (Growth arrest special 5) expression is significantly decreased in ATC tissue samples in comparison to benign tissue samples. In this study, the expression of lncRNA GAS5 showed significant association with TNM staging, lymph node metastasis, and the several cancer foci of thyroid cancer. Therefore, GAS5 may be correlated with diagnosis and prognosis of thyroid cancer [12]. The molecular mechanism of cancer caused by downregulation of GAS5 still remains unknown. Downregulation of GAS5 probably increases the expression of cycling-dependent kinase 6 (CDK6) and consequently promotes cell cycle progression and proliferation [45].

The differentiation grade of neoplastic cells is a common evaluation of tumor malignancy, being lost in the advanced and aggressive types of thyroid cancer such as ATC. In thyroid cancer, the presence of differentiated cells is very important; because of their unique ability to concentrate iodine can be used for radioiodine therapy after surgery, to remove residual and/or metastatic cancer cells. ATC lack this differentiated function and therefore radioiodide therapy is not effective against it. Klhl14-AS (Kelch like family member 14) expression at early stages of thyroid development suggested a potential role in cellular and molecular mechanisms regulating cell differentiation and proliferation. Credendino and colleagues showed that the expression of Klhl14-AS is downregulated in different kinds of thyroid cancer, especially ATC tissues and cell lines. Downregulating Klhl14-AS expression in normal thyrocytes decreased the expression of thyroid differentiation markers and apoptosis and increased cell viability. These effects are mediated by the binding of Klhl14-AS to miR-182-5p and miR-20a-5p, which silenced PAX8 and BCL2, both important factors of thyroid differentiation and homeostasis [17]. Therefore, Klhl14-AS with potential tumor suppression activity may involve in thyroid differentiation and carcinogenesis and can be an effective target for ATC molecular therapy.

Song et al.. indicated that thyroid cancer tissues including ATC samples have a lower expression of LINC00312 (long intergenic non-protein coding RNA 312), also known as NAG7, than adjacent normal tissues. Low expression of LINC00312 was found in patients with larger tumor, lymphatic metastasis, and advanced TNM stages (III and IV). Conversely, miR-197-3p is highly expressed in patients with such clinicopathological characteristics. Their findings demonstrated that LINC00312 overexpression could reduce the proliferation, migration, and invasion of ATC cell line by regulating the expression of miR-197-3p and its target p120 [21]. Evidences showed that miR-197 directly targets p120 through binding to the 3′UTR of p120 mRNA. Also, decreased p120 expression is associated with an increased risk of cancer cell migration and invasion via its impact on cadherin stability and turnover [46, 47]. In ovarian and pancreatic cancer cells, increased expression of miR-197-3p induces invasion and migration [46, 48]. In another study, Min and colleagues showed that LINC00312 expression is decreased in ATC cell line compared with normal thyroid follicular epithelial cells. LINC00312 knockdown with siRNA increased the proliferation and invasion of ATC cell line. Conversely, overexpression of LINC00312 decreased cell proliferation and invasion in vitro, and tumorigenicity in vivo. LINC00312-mediated tumor suppression in ATC cells may occur through suppression of PI3K/AKT signaling pathway and MMP9 gene expression [22]. Among the matrix metalloproteinases, MMP-9 has an important role not only in extracellular matrix degradation during tissue remodeling, but also in pathological processes such as invasion and metastasis of tumor cells and overexpresses in thyroid cancer cells [49–51].

MIR22HG (MIR22 host gene) is a tumor suppressor lncRNA involved in proliferation and progression of several types of cancer such as lung cancer. MIR22HG is downregulated and associated with patient survival in lung cancer. Decreased MIR22HG expression is associated with increased expression levels of MET and p21 oncogenes and cancer cell survival via its impact on YBX1 protein stability [52]. Qin and colleagues showed that MIR22HG is downregulated in thyroid cancer including ATC tissues by analyzing TCGA database. They found that the lower expression levels of MIR22HG is correlated to higher age, lymph node metastasis, and advanced TNM stages as well as higher MIR22HG expression is associated with longer overall and disease-free survival time in thyroid cancer patients. Bioinformatics analysis indicated that MIR22HG is involved in apoptosis and cell cycle processes, transcription and mRNA splicing regulation as well as and Hippo signaling pathway in thyroid cancer [27].

Recent findings have shown that SNHG15 (Small nucleolar RNA host gene 15) is involved in proliferation, migration, and invasion of thyroid cancer and correlated with age, clinicopathological characteristics, and disease-free survival [53]. Liu et al.. showed that there is a negative association between the expression of SNHG15 and miR‐510‐5p expression in ATC cell line. In addition, miR‐510‐5p increased ATC cell line proliferation, migration, and invasion through downregulating SNHG15 [25].

### Oncogenic lncRNAs in ATC

Kim and colleagues indicated that LOC100507661 expression is significantly increased in thyroid cancer tissues compared to paired adjacent normal tissues and suggested that it probably serves as an oncogene in thyroid cancers [11]. Furthermore, they showed that the expression level of LOC100507661 is higher in papillary thyroid cancer (PTC) and ATC cell lines with metastasis to lymph node or *BRAF*^V600E^ mutation [11]. They also revealed that vector-mediated overexpression of LOC100507661 in ATC cells induced cell proliferation, migration, and invasion [11]. Currently, there is no information about the functional mechanisms of LOC100507661. However, because of the lower expression level in thyroid than in other tissues and higher expression level in fetal thyroid it may play an important role in thyroid development and tumorigenesis [11].

Evidences have shown that H19 can function either as a tumor suppressor or as a tumor promoter, depending on the type, stage, and genetic background of cancer cells. Zhang et al. showed that H19 is upregulated in ATC tissues. In this study, targeted inhibition of H19 with siRNA decreased proliferation, migration, and invasion and increased apoptosis in ATC cell line, 8505C, in vitro and inhibited tumorigenesis and metastasis in vivo. Therefore, the H19 may be an effective target for ATC molecular therapy [29].

UCA1 (Urothelial carcinomaassociated 1) serves as an oncogenic lncRNA [54] and is originally recognized in bladder transitional cell carcinoma [55]. Studies have shown that this lncRNA promotes tumor progression in a wide variety of cancers such as prostate cancer, gastric cancer, and esophageal cancer [56–58]. Wang et al. confirmed the oncogenic function of UCA1 in the progression of ATC. They showed that the expression levels of UCA1 increased in ATC tissue samples and cell lines. The results of this study indicated that UCA1 increases cell viability, proliferation, migration, and invasion in ATC in vitro and in vivo through binding to miR135a and regulation of cmyc expression. Several studies demonstrated that the expression of c-myc is upregulated in ATC, and targeting of cmyc may be a potential treatment for ATC [14].

Wang and colleagues examined a number of thyroid cancers which included 28 PTC and 10 ATC tissue samples using RT2 Profiler PCR Array Human Cancer Pathway Finder consisting of 84 lncRNAs to determine the specific lncRNAs that are overexpressed during thyroid cancer progression. They indicated that there are a significant number of lncRNAs that are expressed at higher levels in ATC compared with PTC such as HOTAIRM1 (Hox antisense intergenic RNA myeloid 1) and PCA3 (Prostate cancer antigen 3). The results of this study revealed that the lncRNAs HOTAIRM1 and PCA3 are upregulated during thyroid cancer development and progression [15]. The molecular mechanism of cancer caused by upregulation of HOTAIRM1 and PCA3 in ATC still remains unknown. However, because of their proven role in the progression of ATC, HOTAIRM1 and PCA3 may be used as effective targets for ATC molecular therapy. Since HOTAIRM1 and PCA3 are upregulated in various cancers [59–62]. These findings suggest that they may function as an oncogene in thyroid cancer, especially ATC. It should be noted that a number of evidences have shown that HOTAIRM1 can function either as a tumor suppressor or as a tumor promoter, depending on the type of cancer [61–63].

HCP5 (HLA complex P5) is dysregulated in a wide variety of cancers and diseases such as osteosarcoma [64], cervical cancer [65], and glioma [66] as well as psoriasis [67] and kawasaki disease [68]. Chen et al. showed that HCP5 is upregulated in ATC tissues. In this study, targeted inhibition of HCP5 with siRNA decreased cell viability and increased apoptosis in ATC cell lines. Mechanistically, HCP5 serves as a sponge for miR-128-3p and regulates the expression of miR-128-3p in ATC cell lines and tissues [16].

Du et al. found that Linc00210 (long intergenic non-protein coding RNA 210) expression is increased in thyroid cancer tissues compared to the normal tissue samples. Overexpression of Linc00210 increased the proliferation, migration, and invasion of ATC cell line. Also, they found that Linc00210 directly bound to miR-195‐5p and regulates the expression of IGF1R and the activation of PI3K/AKT signaling in ATC cell line. IGF/IGFR signaling pathway contributes to tumorigenesis and also drug resistance in thyroid cancer via activation of PI3K/AKT signaling pathway, leading to the expression of pro‐survival genes and increased cell proliferation. Inhibition of IGF1R can decrease the responsiveness of thyroid cancer cells to growth factors [18].

Hou and colleagues revealed that TNRC6C-AS1 (TNRC6C antisense RNA 1) is upregulated in ATC tissues compared to adjacent normal tissues and ATC cell lines compared with normal human thyroid epithelial cells. In this study, targeted inhibition of TNRC6C-AS1 with shRNA decreased cell proliferation, migration, and invasion in ATC cell lines through downregulation of UNC5B expression and inhibited thyroid tumorigenesis in vivo. TNRC6C-AS1 serves as a competing endogenous RNA, (ceRNA) and upregulates the expression of UNC5B by sponging miR-129-5p in ATC cell lines. In addition, they found that the expression level of TNRC6C-AS1 in ATC cell lines is much higher than in follicular thyroid cancer (FTC) cell line [19]. Evidences showed that UNC5B, a target gene of miR-129-5p, act as a tumor suppressor in various cancers such as bladder cancer and pancreatic cancer. However, their findings came to opposite conclusion that UNC5B knockdown decreased thyroid cancer progression [69, 70].

Li et al. found that AK139328 expression is upregulated in thyroid cancer tissues including PTC and FTC as well as ATC cell line. Based on their findings, upregulating AK139328 expression increases cell viability, invasion, and cell cycle progression in ATC cell line opposing to the effect in groups with AK139328 knockdown using siRNA [20]. Identifying the molecular mechanism in which AK139328 serves as an oncogenic lncRNA in ATC as well as the therapeutic efficiency by targeted inhibition of AK139328 demands further examination. However, evidences showed that the silencing AK139328 can inhibit necrosis and caspase-3 activities following ischemia/reperfusion treatment [71].

Liu et al. demonstrated that thyroid cancer tissues have a higher expression of XIST (X-inactive specific transcript) than adjacent normal tissues. High expression of XIST was found in patients with larger tumor diameters, and advanced TNM stages (III and IV). They revealed that XIST acts as a ceRNA for miR-34a via sponging miR-34a, competing with hepatocyte growth factor receptor (MET) for miR-34a binding, and decreasing ATC cell lines proliferation in vitro and tumor growth in vivo. Moreover, targeted inhibition of XIST could decrease the protein levels of MET, and the phosphorylation of PI3K and AKT [23]. Garcia and colleagues have recently indicated that MET overexpression and activation can increase cell invasion in ATC-derived cell line [72].

Chen et al.. revealed that SNHG7 (Small nucleolar RNA host gene 7) is overexpressed in thyroid cancer tissues by analyzing of The Cancer Genome Atlas (TCGA) datasets. The high expression levels of SNHG7 are correlated with advanced stages (III and IV) and shorter survival times in of thyroid cancer patients. Their funding showed that SNHG7 knockdown inhibits the cell proliferation and cell cycle progression of ATC cell line in vitro [24]. The molecular mechanism of ATC caused by upregulation of SNHG7 still remains unknown.

Evidences have shown that ZFAS1 (ZNFX1 antisense RNA 1) can function either as a tumor suppressor or as a tumor promoter, depending on the type of cancer cell. For example, ZFAS1 was first introduced as a tumor suppressor in breast cancer. But in hepatocellular carcinoma ZFAS1 serves as an oncogene and promotes cancer metastasis by regulating the expression of related genes such as MMP14 and MMP16 [73]. Han et al. revealed that ZFAS1 is highly expressed in thyroid cancer tissues, including ATC samples, compared to normal tissue samples by analyzing RNA-seq dataset of thyroid cancer from TCGA. ZFAS1 overexpression is positively associated with clinicopathological characteristics and poor prognosis in thyroid cancer. Targeted inhibition of ZFAS1 with siRNA decreased proliferation, and cell cycle progression in ATC cell line [26].

NEAT1 (Nuclear paraspeckle assembly transcript 1) acts as a ceRNA involved in several types of cancer. A number of evidences showed that NEAT1 has an important role in cancer chemoresistance. In addition, overexpression of NEAT1 is correlated with thyroid cancer progression by sponging miR214 and downregulation of NEAT1 is associated to suppression of PTC progression via the miR1295p/KLK7 axis [74]. Yan and colleagues indicated that NEAT1 is overexpressed in ATC cell lines and tissue samples, and NEAT1 silencing resulted in decreased cisplatinresistance of ATC through the regulating of miR95p/SPAG9 axis in vitro and in vivo [29]. Identifying the molecular mechanism in which NEAT1 serves as an oncogenic lncRNA in ATC as well as the therapeutic efficiency by targeted inhibition of NEAT1 demands further investigation.

### LncRNAs related to cancer stem cell properties in ATC

Recent developments in cellular and molecular biology provide growing evidence that a specific subpopulation of tumor cells plays an important role in tumor initiation, invasion, and resistance to treatment in thyroid cancers, especially in ATC [30, 75–78]. These cells named tumor-initiating cells (TICs) or CSCs, have the stem cell-like properties such as the ability of self-renewal, colony formation, and EMT as well as induce resistance to chemotherapy and radiation therapy and telomerase reverse transcriptase (TERT) expression [30, 78–80]. EMT has a crucial role in tumor progression and invasion. During EMT, cells lose cellular adhesion and polarity and acquire an invasive phenotype [78, 81]. Recent studies have indicated that lncRNAs play an essential role in the maintenance of CSCs and can modulate CSCs properties [82–84]. However, studies that have examined only the role of lncRNAs in thyroid CSCs, particularly in ATC, are scarce and in many cases, lncRNAs associated with CSCs properties overlap with their oncogenic or tumor suppressive function (Table 2).

**Table 2:** LncRNAs and their roles in ATC

LncRNA	Tumor suppressor	Oncogene	CSC properties
AK139328	−	+	N/D
BANCR	−	+	+
CASC2	+	−	N/D
FOXD2-AS1	−	+	+
GAS5	+	−	N/D
H19	−	+	N/D
HCP5	−	+	N/D
HOTAIR	+	−	+
HOTAIRM1	−	+	N/D
KLHL14-AS	+	−	N/D
Linc00210	−	+	N/D
LINC00312	+	−	N/D
LOC100507661	−	+	N/D
MALAT1	−	+	+
MANCR	−	+	+
MIR22HG	+	−	N/D
NEAT1	−	+	N/D
PAR5	+	−	+
PCA3	−	+	N/D
PTCSC3	+	−	+
PVT1	−	+	+
ROR	−	+	+
SNHG15	+	−	N/D
SNHG7	−	+	N/D
TNRC6C-AS1	−	+	N/D
TUG1	−	+	+
UCA1	−	+	N/D
XIST	−	+	N/D
ZFAS1	−	+	N/D

N/D, not detected; BANCR, BRAF-activated non-protein coding RNA; CASC2, cancer susceptibility candidate 2; CSC, cancer stem cell; FOXD2-AS1, FOXD2 adjacent opposite strand RNA 1; GAS5, growth arrest special 5; HCP5, HLA complex P5; HOTAIR, HOX transcript antisense RNA; HOTAIRM1, hox antisense intergenic RNA myeloid 1; Klhl14, kelch like family member 14; Linc00210, long intergenic non-protein coding RNA 210; LINC00312, long intergenic non-protein coding RNA 312; LncRNA, long noncoding RNA, MALAT1: metastasis-associated lung adenocarcinoma transcript 1; MANCR, mitotically associated long non coding RNA; MIR22HG, MIR22 host gene; NEAT1, nuclear paraspeckle assembly transcript 1; PAR5, Prader Wili/Angelman region RNA 5; PCA3, prostate cancer antigen 3; PTCSC3, papillary thyroid carcinoma susceptibility candidate 3; PVT1, plasmacytoma variant translocation 1; ROR, regulator of reprogramming; SNHG15, small nucleolar RNA host gene 15; SNHG7, small nucleolar RNA host gene 7; TNRC6C-AS1, TNRC6C antisense RNA 1; TUG1, taurine up-regulated gene 1;UCA1, urothelial carcinoma-associated 1; XIST, X inactive specific transcript; ZFAS1, ZNFX1 antisense RNA 1

ROR (Regulator of reprogramming) was first detected in induced pluripotent stem cells (iPSCs) [85, 86]. Hardin et al.. identified ROR in ATC cell line THJ-16T and CSC derived from the same ATC cell line by reverse transcription polymerase chain reaction (RT-PCR). According to their results, the expression level of ROR was higher in the CSC clone compared to the parental ATC cell line [30]. Recent research has suggested that ROR may exert its effect via modulation of the key pluripotency genes including OCT4, SOX2, and Nanog [30, 87, 88]. In another study, Hardin and colleagues focused on thyroid cancer stem-like cell exosomes which transfer lncRNAs involved in EMT. Exosomes are small (30–150 nm) membranous vesicles secreted by most cells that have an important role in cell-cell communication. They demonstrated that CSC exosomes transfer lncRNA ROR, to induce EMT in ATC cells. They also determined the lncRNA expression levels in CSC clones of ATC line compared to the parental line and found that MALAT1 and ROR are upregulated and HOTAIR (HOX transcript antisense RNA) and PVT1 (Plasmacytoma variant translocation 1) are downregulated. In addition, stem cell marker SOX2 and EMT marker SLUG had increased expression in CSC clonal lines compared to their parental ATC line. Evidences showed that MALAT1 is increased in PTC but significantly decreased in ATC and PVT1, HOTAIR, and ROR are downregulated in ATC compared to PTC [30]. The molecular mechanism that causes differences in the expression of these lncRNAs in ATC with PTC still remains unclear.

Some studies have shown that PVT1 is involved in poor prognosis of cancers [89, 90]. Zhou et al.. demonstrated that PVT1 expression is increased in ATC tissues and cell line compared with normal controls [31]. They indicated that the suppression of PVT1 causes cell cycle arrest at G_0_/G_1_ phase and significantly decreases cyclin D1 expression, thyroid stimulating hormone receptor (TSHR) expression, and the proliferation of thyroid cancer cells in ATC cell line [31]. In addition, Zhou and colleagues demonstrated that PVT1 could be enriched by enhancer of zeste homolog 2 (EZH2) [31], a marker of an advanced metastatic disease in numerous types of cancer including ATC [91–94], which may also contribute to the regulation of TSHR expression [31]. The authors finally proposed that PVT1 may contribute to pathogenesis of thyroid cancer through EZH2 recruitment and TSHR expression regulation [31].

MALAT1 (Metastasis-associated lung adenocarcinoma transcript 1) expression is associated with the tumor development, invasion, metastasis, and outcome in different types of cancer [95]. In addition, several reports have indicated that MALAT1 is expressed in CSCs of various cancers and has been linked to EMT [96, 97], a CSC feature [98–101]. Zhang et al.. analyzed 195 samples of normal/benign thyroid tissues as well as malignant thyroid neoplasms [100]. They indicated that expression of MALAT1 decreased during progression from normal thyroid tissue to ATC, but it is increased in PTC as compared to normal thyroid tissues. Based on their results, the greatest expression level occurs in PTC and the lowest expression level belongs to ATC in comparison to normal thyroid tissues [100]. Furthermore, they demonstrated that MALAT1 expression was elevated after inducing EMT in PTC cell line TPC-1 by tissue growth factor beta (TGF-β) [100]. Huang and colleagues also confirmed the upregulation of MALAT1 in differentiated thyroid cancers in comparison with adjacent normal thyroid tissues [102]. In addition, they showed that MALAT1 significantly increased in FTC and ATC cell lines compared to human normal breast epithelial cells, as the control with the highest expression level occurred in FTC and then in ATC cells [102]. However, in contrast to Zhang et al..’s study [100], the MALAT1 expression level in ATC cell lines were not compared to normal thyroid cells in this study [102]. According to their results, MALAT1 exerts its effect by the upregulation of IQGAP1 [102], a key protein in regulating the cell adhesion and movement, which is also involved in EMT [102–105]. In addition, Huang and colleagues indicated that the overexpression of MALAT1 and IQGAP1 is correlated with the proliferation and invasion of thyroid cancer cells [102]. Another possible mechanism which MALAT1 may implicate its effect is the modulation of EZH2. EZH2 contributes to tumorigenesis and cancer progression, often through inactivation of tumor suppressor genes [31, 106]. According to our knowledge, the association between MALAT1 and EZH2 in ATC has not been evaluated yet. Nevertheless, several studies demonstrated that MALAT1 augments the oncogenic activities of EZH2 in different types of cancer [106–108]. As a result, EZH2 may be the common pathway through which PVT1 and MALAT1 apply their effects as an oncogene in ATC. In our previous study, we used 3D *in vitro* ATC model to determine the effect of BI-847,325 anticancer drug on MAPK signaling pathway via evaluating of the molecular mechanisms of MALAT1-mediated genes’ regulation in ATC cell lines. The result of that study was the first report of MALAT1’s molecular function in ATC and suggested that MALAT1 could be effective on cell cycle and apoptosis by regulating the expression of MALAT1-mediated cell cycle- and apoptosis-associated genes including Mcl1, miR-363-3p, and cyclin D1 [34]. In addition, Gou and colleagues showed that MALAT1 is upregulated in ATC cell lines. In this study, targeted inhibition of MALAT1 with siRNA decreased proliferation, migration, and invasion and increased apoptosis and autophagy formation in ATC cell line *in vitro* and inhibited tumorigenesis and metastasis in murine xenograft model through regulation of miR-200a-3p and FOXA1 expression. Therefore, the MALAT1 as a ceRNA may be an effective target for ATC molecular therapy [35]. Put together, these results suggest that MALAT1 plays an essential role in the pathogenesis of thyroid cancers and may have a significant role in anaplastic thyroid CSCs. However, further studies are needed to define its exact role in ATC.

Liu and colleagues used ATC cell line to determine the effect of flavonoid luteolin, a natural antioxidant, as anticancer drug on lncRNA BANCR (BRAF-activated non-protein coding RNA) and its downstream genes. They showed that luteolin decreases cell growth and colony formation ability and increases cell cycle arrest at G_1_/S phases through modulation of BANCR/TSHR/CCND1 signaling pathway [36].

FOXD2-AS1 (FOXD2 adjacent opposite strand RNA 1) is an oncogenic lncRNA that is overexpressed in bladder cancer and promoted progression and recurrence via forming a positive feedback loop with AKT and E2F transcription factor 1 (E2F1) [109]. In addition, increased expression of this lncRNA in gastric cancer patients can predict poor prognosis and promotes tumorigenesis by epigenetically silencing EphB3 (EPH receptor B3) via EZH2 and LSD1, also known as KDM1A (lysine demethylase 1A) [110]. Liu et al. indicated that FOXD2-AS1 expression is upregulated in ATC tissues compared with the adjacent normal samples, through analyzing RNA-seq dataset of thyroid cancer from TCGA database. FOXD2-AS1 overexpression is correlated with advanced TNM stags, recurrence status, and disease-free survival in thyroid cancer patients. Also, they found that the expression level of FOXD2-AS1 is increased in ATC cell lines compared with normal thyroid follicular epithelial cells. Their results showed that FOXD2-AS1 acts as a ceRNA for miR-7-5p, upregulating the expression of TERT, which increases the CSCs properties and anoikis resistance in thyroid cancer cells [111].

The expression of PTCSC3 (Papillary thyroid carcinoma susceptibility candidate 3) is strictly thyroid-specific and is downregulated in thyroid cancer. Wang et al.. indicated that PTCSC3 has a role in ATC by regulation STAT3/INO80 pathway. They reported that downregulation of PTCSC3 increases INO80 and MDR-1 expression through positively regulating STAT3, and thereby increasing drug resistance of ATC to Doxorubicin. Also, the results of this study indicated that INO80 influences on pluripotent state of CSCs by positively regulating ALDH and CD^133^ as stem cell markers in ATC tissues and cell line [38]. Fan and colleagues indicated the effect of PTCSC3 on cell growth, cell cycle, and apoptosis in ATC cell line. In this study, PTCSC3 is investigated as a competing endogenous RNA (ceRNA) for miR-574-5p [39]. Currently, there are no evidences about the role of miR574-5p in ATC. However, several studies demonstrated that miR-574-5p plays a role in proliferation, anchorage-independent growth of cancer cells, resistance to chemotherapy, and poor prognosis in patients with various cancers [112, 113].

Lu and colleagues revealed that higher MANCR (Mitotically associated long non coding RNA), also known as LINC00704, expression levels are associated with shorter overall survival time in thyroid cancer patients. In this study, targeted inhibition of MANCR with siRNA decreased EMT through upregulating E-cadherin and downregulating N-cadherin and β-catenin expression, proliferation, and colony formation ability, as well as increased G_0_/G_1_ cell cycle arrest and apoptosis in ATC cell line in vitro [40].

Studies showed that PAR5 (Prader Wili/Angelman region RNA 5) is involeved in glioblastoma multiforme and its downregulation directly correlated with cancer progression [114]. Pllecchia et al.. assessed lncRNA expression profile of ATC tissues in comparison with normal thyroid samples through microarray. They identified 19 upregulated and 28 downregulated lncRNAs in ATC samples. Then, they evaluated the expression of three upregulated (MIAT, BCYRN1, BIC) and three downregulated (RMST, PAR5, IPW) lncRNAs to validate the microarray results. Subsequently, they found that PAR5 significantly and specifically downregulated in ATC. The restoration of PAR5 decreased colony formation ability, proliferation, and migration of ATC cell lines indicating that its downregulation contributes to ATC progression. They also showed that PAR5 interacts with EZH2 in ATC cell lines, reducing EZH2 protein levels and its binding on the E-cadherin promoter, relieving E-cadherin, as a key marker in switching EMT, from the negative regulation by EZH2 [41].

First, TUG1 (Taurine up-regulated gene 1) was identified as a potential lncRNA involved in mouse retinal development. Currently, the dysregulation of TUG1 has been reported in several cancers. Evidences revealed that TUG1 serves as ceRNA by sponging the miRNA or working via binding to polycomb repressive complex 2 (PRC2) [115]. Lei et al. indicated that TUG1 is an oncogenic lncRNA in thyroid cancer, and it is overexpressed in ATC cell lines compared with human normal breast epithelial cells. TUG1 can increase the proliferation, invasion, migration, and EMT of ATC cells. Their findings showed that miR-145 is a potential target of TUG1, and miR-145/ZEB1 signaling pathway mediates the function of TUG1 in ATC cells [42].

## Conclusions

In addition to genetic and epigenetic changes, other biological mechanisms including lncRNAs play a crucial role in regulation of approximately all steps of cancer progression. LncRNAs can affect on molecular mechanisms involved in the development and progression of ATC including the regulation of epigenetic factors and expression of genes and miRNAs associated with proliferation, invasion, metastasis, cell cycle, apoptosis as well as stem cell-like properties of CSCs such as EMT and colony formation (Table 3). For most lncRNAs, only a single experiment has evaluated the expression profile in ATC tissues and/or cells. Therefore, there is no way for comparison and verification studies. Consequently, further functional studies and expression profiling in larger sample sizes are needed to resolve this limitation and identify novel and valid biomarkers.

**Table 3:** The possible mechanisms of lncRNAs in ATC

LncRNA	Cell proliferation/viability/growth	Cell cycle	Invasion	Cellular function				
				Migration	Apoptosis	Differentiation	Autophagy	CSC properties
LOC100507661	√		√	√				
GAS5	√	√						
H19	√		√	√	√			
UCA1	√		√	√				
HCP5	√				√			
KLHL14-AS	√				√	√		
Linc00210	√		√					
TNRC6C-AS1	√		√	√				
AK139328	√	√	√					
LINC00312	√		√	√				
XIST	√							
SNHG7	√	√						
SNHG15	√		√	√				
ZFAS1	√	√						
CASC2	√	√						
ROR								√
PVT1	√	√						√
HOTAIR								√
MALAT1	√		√	√	√		√	√
BANCR	√	√						√
FOXD2-AS1								√
PTCSC3	√	√			√			√
MANCR	√	√			√			√
PAR5	√							√
TUG1	√		√	√				√

lncRNAs such as MIR22HG, HOTAIRM1, and PCA3 are involved in the development, progression, and prognosis of ATC, their function in different cellular processes have not yet been determined

BANCR, BRAF-activated non-protein coding RNA; CASC2, cancer susceptibility candidate 2; CSC, cancer stem cell; FOXD2-AS1, FOXD2 adjacent opposite strand RNA 1; GAS5, growth arrest special 5; HCP5, HLA complex P5; HOTAIR, HOX transcript antisense RNA; Klhl14, kelch like family member 14; Linc00210, long intergenic non-protein coding RNA 210; LINC00312, long intergenic non-protein coding RNA 312; LncRNA, long noncoding RNA, MALAT1: metastasis-associated lung adenocarcinoma transcript 1; MANCR, mitotically associated long non coding RNA; PAR5, Prader Wili/Angelman region RNA 5; PTCSC3, papillary thyroid carcinoma susceptibility candidate 3; PVT1, plasmacytoma variant translocation 1; ROR, regulator of reprogramming; SNHG15, small nucleolar RNA host gene 15; SNHG7, small nucleolar RNA host gene 7; TNRC6C-AS1, TNRC6C antisense RNA 1; TUG1, taurine up-regulated gene 1; UCA1, urothelial carcinoma-associated 1; XIST, X inactive specific transcript; ZFAS1, ZNFX1 antisense RNA 1

Analysis of lncRNAs in ATC can be an interesting field, which will lead to identification of novel diagnosis and prognosis markers, because of the advantages such as noninvasiveness and easy access. At present, only a few parts of lncRNAs have been identified in ATC that may serve as prognosis markers such as GAS5, MIR22HG, and CASC2. Also, because of the dysregulation of Klhl14-AS, HOTAIRM1, and PCA3 during ATC development and progression, they may act as therapeutic targets. The measurement of lncRNAs in blood and fine needle aspiration biopsy of ATC patients can be useful as an adjunct to improving diagnosis and prognostication. However, further studies are required to determine the exact function of lncRNAs in the development and progression of ATC, which can contribute to the identification of novel and valid biomarkers, as well as possible therapeutic targets in ATC.

## Data Availability

Not applicable.

## Abbreviations

AKT: AKT serine/threonine kinase
ALDH1: aldehyde dehydrogenase 1
ATC: anaplastic thyroid cancer
BANCR: BRAF-activated non-protein coding RNA
BCL2: BCL2 apoptosis regulator
BCYRN1: brain cytoplasmic RNA 1
BRAF: B-raf proto-oncogene
CASC2: cancer susceptibility candidate 2
CCND1: cyclin D1
CDK6: cycling-dependent kinase 6
ceRNA: competing endogenous RNA
c-myc: avian myeocytomatosis virus oncogene cellular homolog
CSC: cancer stem cell
EMT: epithelial to mesenchymal transition
EZH2: enhancer of zeste homolog 2
FOXA1: forkhead box protein A1
FOXD2-AS1: FOXD2 Adjacent Opposite Strand RNA 1
FTC: follicular thyroid cancer
GAS5: growth arrest special 5
HCP5: HLA complex P5
HOTAIR: HOX transcript antisense RNA
HOTAIRM1: HOX antisense intergenic RNA myeloid 1
IGF: insulin growth factor
IGF1R: insulin like growth factor 1 receptor
INO80: INO80 complex ATPase subunit
iPSC: induced pluripotent stem cell
IPW: imprinted in prader-willi syndrome
IQGAP1: Ras GTPase-activating-like protein
Klhl14: kelch like family member 14
KLK7: kallikrein related peptidase 7
Linc00210: long intergenic non-protein coding RNA 210
LINC00312: long intergenic non-protein coding RNA 312
LncRNA: long noncoding RNA
MALAT1: metastasis-associated lung adenocarcinoma transcript 1
MANCR: mitotically associated long non coding RNA
MAPK: mitogen-activated protein kinase
Mcl1: myeloid cell leukemia 1
MDR-1: multidrug resistance protein 1
MET: MET protooncogene
MIAT: myocardial infarction associated transcript
miR: microRNA
MIR22HG: MIR22 host gene
miRNA: microRNA
MMP16: matrix metallopeptidase 16
cRNA: noncoding RNA
NEAT1: nuclear paraspeckle assembly transcript 1
OCT4: octamer-binding transcription factor 4
PAR5: Prader Wili/Angelman region RNA 5
PAX8: paired box 1
PCA3: prostate cancer antigen 3
PI3K: phosphatidylinositol-3-kinas
piRNA: piwi-interacting RNA
PRC2: polycomb repressive complex 2
PTC: papillary thyroid cancer
PTCSC3: papillary thyroid carcinoma susceptibility candidate 3
PVT1: plasmacytoma variant translocation 1
RAS: RAS proto-oncogene
RMST: rhabdomyosarcoma 2 associated transcript
RNA-seq: RNA sequencing
ROR: regulator of reprogramming
RT-PCR: reverse transcription polymerase chain reaction
ShRNA: short hairpin RNA
siRNA: small interfering RNA
SNHG15: small nucleolar RNA host gene 15
SNHG7: small nucleolar RNA host gene 7
SOX2: SRY-box transcription factor 2
SPAG9: sperm associated antigene 9
STAT3: signal transducer and activator of transcription 3
TCGA: the cancer genome atlas
TERT: telomerase reverse transcriptase
TGF-β: tissue growth factor beta
TIC: tumor-initiating cell
TNM: tumor-node-metastasis
TNRC6C-AS1: TNRC6C antisense RNA 1
TSHR: thyroid stimulating hormone receptor
TUG1: taurine up-regulated gene 1
UCA1: urothelial carcinoma-associated 1
UNC5B: unc-5 netrin receptor B
XIST: X inactive specific transcript
ZEB1: zinc finger E-box binding homeobox 1
ZFAS1: ZNFX1 antisense RNA 1
